# Metacognitive awareness in the sound-induced flash illusion

**DOI:** 10.1098/rstb.2022.0347

**Published:** 2023-09-25

**Authors:** Randolph Maynes, Ryan Faulkner, Grace Callahan, Callie E. Mims, Saurabh Ranjan, Justine Stalzer, Brian Odegaard

**Affiliations:** ^1^ University of Florida, 945 Center Drive, Gainesville, FL 32603, USA; ^2^ Psychology Department, University of South Alabama, Mobile, 36688, AL, USA

**Keywords:** multisensory integration, metacognition, sound-induced flash illusion

## Abstract

Hundreds (if not thousands) of multisensory studies provide evidence that the human brain can integrate temporally and spatially discrepant stimuli from distinct modalities into a singular event. This process of multisensory integration is usually portrayed in the scientific literature as contributing to our integrated, coherent perceptual reality. However, missing from this account is an answer to a simple question: how do confidence judgements compare between multisensory information that is integrated across multiple sources, and multisensory information that comes from a single, congruent source in the environment? In this paper, we use the sound-induced flash illusion to investigate if confidence judgements are similar across multisensory conditions when the numbers of auditory and visual events are the same, and the numbers of auditory and visual events are different. Results showed that congruent audiovisual stimuli produced higher confidence than incongruent audiovisual stimuli, even when the perceptual report was matched across the two conditions. Integrating these behavioural findings with recent neuroimaging and theoretical work, we discuss the role that prefrontal cortex may play in metacognition, multisensory causal inference and sensory source monitoring in general.

This article is part of the theme issue ‘Decision and control processes in multisensory perception’.

## 1. Introduction

Metacognition has previously been defined as the capacity for ‘thinking about thinking’ [1] and perceptual metacognition can be defined as the capacity to monitor the quality and fidelity of one's own perceptions. Studies now provide various behavioural and computational tools to measure perceptual metacognition [2–5], reveal the neural correlates that support this ability [6–11], and demonstrate how perceptual confidence and perceptual accuracy dissociate in specific situations [12–16]. However, the vast majority of research on perceptual metacognition focuses on the *visual* modality alone, and specifically, visual confidence judgements [17]. As others have noted, little is currently known about how our sense of perceptual metacognition extends to multisensory paradigms, with sensory stimulation in two or more sensory modalities [18]. Thus, to better understand what metacognition is, how it functions, and what adaptive purposes it may serve, it is necessary to further explore the role that it plays in monitoring multisensory representations of the external world.

Recent theoretical accounts of metacognition posit that it may play a role in distinguishing between real and imagined stimuli [19], and help facilitate ‘perceptual reality monitoring’ [20] to make accurate inferences about which sources give rise to which sensory stimuli [21]. Interestingly, the process of inferring which external sources in the world give rise to specific sensations is thought to be central to causal inference in multisensory perception [22,23], as the brain must determine if a single source in the environment is producing stimulation in two or more modalities, or if separate sources in the environment are giving rise to multiple sensory signals. If metacognition facilitates our capacity to distinguish between what is real and what is not, might it also help us distinguish between different types of multisensory information in the world?

### (a) Potentially, different types of multisensory experiences come in (at least) three different forms

The first type of multisensory experience is that of a *congruent* multisensory signal. Congruent multisensory signals can be defined by a single source in the environment giving rise to sensations in two or more modalities at the same time. For instance, when you talk to another person, you see their lips move and hear the sound of their voice, and this information arises from one source. Contrasting congruent multisensory signals are *integrated* multisensory signals. Integrated signals occur when distinct sources produce conflicting sensory information (e.g. visual and auditory information), but the brain *infers* that these signals originated from a single source, and combines them into a unique percept.

Examples of this include multisensory illusions such as spatial ventriloquism [24,25], temporal ventriloquism [26,27] and the McGurk effect [28], among others.

Lastly, *segregated* multisensory signals occur when stimulation occurs in two or more sensory modalities, and the brain infers that separate sources give rise to each signal. Considering these different types of multisensory experiences, one can ask: can metacognition help us distinguish between congruent and integrated (illusory) multisensory experiences? And can it do so when our perceptual reports about what we experience are identical across two or more experimental conditions [18]?

It is interesting to consider what a preliminary hypothesis should be when comparing confidence in congruent multisensory perception with confidence in integrated multisensory perception. Over the last 40 years, a tremendous amount of research has emphasized benefits in multisensory integration. One primary benefit comes in reducing and resolving perceptual ambiguity [29], as many studies attest to the finding that when stimuli are integrated from discrepant sources, the resulting representation is more precise than the pre-existing unisensory representations [30–32]. Further, past research has provided evidence of ‘superadditivity’ in brain responses to integrated multisensory stimuli, showing that neural responses to multisensory stimuli that are somewhat coincident in either space or time are often larger than the sum of unimodal responses, especially for weak stimuli [33–36]. But superadditivity may not be a hallmark of all multisensory interactions [37], and while it remains possible that the process of integrating stimuli could contribute a unique signal that leads to stronger metacognition for integrated stimuli over congruent stimuli, this seems unlikely. Perhaps confidence judgements for integrated and congruent multisensory stimuli are similar? If it is difficult for observers to tell integrated and congruent multisensory signals apart, this seems possible. However, research demonstrates that enhanced brain responses can occur for congruent multisensory information [38], which could lead to higher confidence compared with integrated signals. Importantly, many forms of integrated multisensory stimulation move estimates *away from the true source of information*. For example, the spatial ventriloquist illusion [24,25], where estimates of auditory stimuli are biased by simultaneous visual stimulation, is an example of how multisensory integration makes perception (in an absolute sense) less veridical than if separate representations were maintained for each sensory modality alone. Thus, while multisensory integration has its benefits, it would seem more optimal for observers to be more confident in congruent multisensory information compared with integrated multisensory information. However, to date, little data exist that speak to the behavioural profile of multisensory confidence judgements [39–41].

In this investigation, we explore whether confidence differs for congruent and integrated multisensory stimulation, and if so, whether it also differs when reports are matched across congruent and integrated trials [18]. We do so by exploiting a well-known example of multisensory integration: the sound-induced flash illusion [42,43]. In the ‘fission’ version of this illusion, if observers are presented with two brief beeps and one visual flash, they often report seeing two visual flashes. In the ‘fusion’ version of this illusion, if observers are presented with one beep and two visual flashes, they sometimes report seeing one visual flash [44,45]. Interestingly, participants' reports of the number of visual flashes in these illusory cases may be equivalent to reports in conditions with congruent audiovisual stimulation, where the number of flashes and beeps is the same. These conditions of distinct-stimulation-but-identical-report in the sound-induced flash illusion allow us to compare whether metacognitive confidence in judgements about the numbers of flashes is different between congruent and integrated stimulation, and to evaluate if confidence is different when the percept (i.e. the number of flashes) is matched across conditions.

Previous research supports the hypothesis that phenomenological distinctions can be made between genuine flashes and illusory flashes [46]. Therefore, even if perceptual reports about the number of flashes are the same across conditions, it seems possible that metacognitive systems may be able to index differences by producing different levels of confidence. In our experiment, on each trial, observers were presented with 0–2 flashes and 0–2 beeps, and on each trial were asked to judge two things: (1) the number of flashes that were presented (or if it was a beep-only trial, the number of beeps), and (2) their confidence in their judgement about the number of flashes. To anticipate, our results showed that the profile of metacognition was marked by higher confidence for congruent stimulation and lower confidence for integrated stimulation, and that even when reports were matched across congruent and integrated trials, confidence was still higher for congruent stimuli. We discuss these results and their implications below.

## 2. Experiment—method

### (a) Participants

Forty-six undergraduate students at the University of Florida (33 women, 13 men, mean age = 19.02 years, s.d. = 3.05) volunteered to participate to earn course credit. Participants began the experimental session by completing an informed consent procedure (IRB no. 201902462, University of Florida). All experimental procedures were conducted in accordance with the Declaration of Helsinki.

### (b) Stimuli and apparatus

Participants were positioned approximately 50 cm away from a CRT monitor and were kept in this position for the entire experiment through the use of a chinrest. The computer volume on our Dell PC was set to 30% of system maximum, and the external speaker volume was set to 100%; this yielded an average of 70 dB when tested with consecutive stimulus presentations. Eight conditions were included in our experiment: four unisensory conditions (1 beep (1B), 2 beeps (2B), 1 flash (1F), and 2 flashes (2F)) and four bisensory conditions, including 1-beep/1-flash (1B1F), 2-beeps/1-flash (2B1F), 1-beep/2-flashes (1B2F) and 2-beeps/2-flashes (2B2F). All flashes were presented for 10 ms; all beeps were also 10 ms in duration. In the 1B1F condition, the beep and flash were presented simultaneously. In the 2B2F condition, the beeps and flashes were presented simultaneously, with a 50 ms gap between the initial beep–flash presentation and the second. In the 1B2F condition, there were 50 ms between flashes, and the beep was presented with the first flash. In the 2B1F condition, there was 50 ms between beeps, and the flash was presented with the first beep.

### (c) Procedure

Participants began our task by reading our consent form and signing to provide written consent. Next, participants reported their sex and age for our records. Then they were asked to adjust the chinrest to a comfortable height. Lastly, participants were provided instructions on how to complete the beep–flash illusion task and began a set of eight practice trials. The practice trials consisted of two trials demonstrating the beep sound that would be used, two trials demonstrating what the flash stimulus on the screen looked like, and four trials providing an example of bisensory trials, combining the beep and flash. For the beep-only practice trials, the participants had to report the number of beeps they heard and their confidence level in their decision. For the flash trials, the participants had to report the number of flashes they saw and their confidence level. For the bisensory practice trials, the participants had to report the number of flashes they perceived and their confidence level.

Following the practice trials, the participants began the actual experiment consisting of 240 psuedorandomly ordered trials from all eight conditions, which were split up into six blocks of 40 trials. Unfortunately, despite using MATLAB's functions to randomize stimuli properly, we failed to randomize the starting seed in the program (using ‘rng shuffle’), and thus, 27 of our 46 participants received the same pseudorandomized order of trials. Participants were allowed to take a break in between each block. As with the practice task, the participants were presented with eight possible conditions, which were pseudorandomly ordered: 1B, 2B, 1F, 2F, 1B1F, 1B2F, 2B1F, 2B2F. Trials were structured so that each began with a white fixation cross in the middle of a black screen for 1000 ms, followed by the presentation of stimuli, and then by a prompt asking for the participant's responses. The white flash was centred on the screen, approximately 4° below fixation. After the stimulus presentation, in the 1F, 2F, 1B1F, 1B2F, 2B1F and 2B2F conditions, participants had to report the number of flashes they perceived and their confidence in that decision for each trial. Confidence was rated on a discrete 1–4 scale, with 1 = not at all confident, and 4 = extremely confident. In the 1B and 2B conditions, they reported the number of beeps they perceived, and their confidence in the decision for each trial. In total, the experiment lasted approximately 40 min on average.

## 3. Results

**Figure 1.**
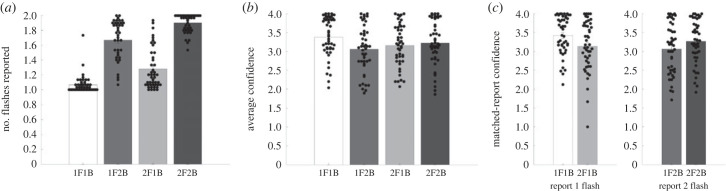
Average behavioural reports across subjects and individual averages in the beep–flash illusion conditions. All error bars represent standard error of the mean. (*a*) The average number of flashes reported across subjects in each condition. (*b*) The average confidence in the type 1 judgement about the number of flashes. (*c*) Average confidence for judgements with the same type 1 Report. The pair of bars in the left part of this panel reflect trials where subjects report perceiving one flash in these two conditions, and the pair in the right of this panel reflect trials where subjects report perceiving two flashes.

As shown in figure 1*a*, we were able to successfully create stimulus conditions that frequently resulted in both ‘fission’ and ‘fusion’ illusions. For example, participants in the 1F2B condition (the ‘fission’ illusion condition) frequently reported two flashes (mean = 1.67, s.d. = 0.26), compared with the 1F1B condition, where they frequently reported one flash (mean = 1.06, s.d. = 0.12). To test whether the difference between conditions was significant, we conducted a Shapiro–Wilk test of normality, which suggested a deviation from normality (*W* = 0.95, *p* = 0.049); therefore, we conducted a Wilcoxon signed-rank test, which indicated that the average number of flashes reported in these two conditions was significantly different (*p* < 0.001). Participants in the 2F1B condition (the ‘fusion’ illusion condition) frequently reported one flash, compared with the 2F2B condition, where they frequently reported two flashes. A Shapiro–Wilk test of normality indicated a deviation from normality (*W* = 0.93, *p* = 0.01); we conducted a Wilcoxon signed-rank test, which indicated that the average number of flashes reported in these two conditions was significantly different (*p* < 0.001).

Next, we plotted the average confidence across our four stimulus conditions (figure 1*b*). On average, confidence was highest in the 1F1B (3.38) and 2F2B conditions (3.22), and lower in the 1F2B (3.06) and 2F1B (3.15) conditions. However, our most important analysis in this project focused on trials where the type 1 report was *matched* between different conditions. Specifically, certain conditions frequently resulted in reports of one flash (1F1B; 2F1B) or two flashes (1F2B; 2F2B). We hypothesized that confidence judgements would be able to distinguish congruent multisensory sensations from illusory multisensory sensations, even when the type 1 report was the same. To answer this question, we first selected all of the trials that resulted in a report of one flash in the 1F1B and 2F1B conditions, and all of the trials that resulted in a report of two flashes in the 1F2B and 2F2B conditions. Then, we computed the average confidence for each subject for these trials (figure 1*c*). As can be seen in the figure, confidence was highest for congruent multisensory trials, and lower for illusory multisensory trials; this was true not only when one flash was reported (*W* = 771; *p* < 0.001), but also when two flashes were reported (*W* = 105; *p* < 0.001).

For unisensory trials, confidence was higher when judging the numbers of beeps compared with the numbers of flashes, which is in line with the general conception of the auditory modality being more precise in the temporal domain [47]. Specifically, confidence for the 1B (mean = 3.71, s.d. = 0.42) and 2B conditions (mean = 3.69, s.d. = 0.43) was higher than average confidence in the 1F (mean = 3.38, s.d. = 0.5) or 2F conditions (mean = 3.21, s.d. = 0.54).

In addition to these analyses of averages within a condition, confidence can also be analysed in terms of correct and incorrect trials within each condition. Within unisensory conditions, confidence was much higher for correct compared with incorrect trials. When computing the average confidence across subjects for unisensory visual trials (after excluding subjects that did not have any incorrect trials), confidence for correct trials in the 1F condition was much higher (mean = 3.42, s.d. = 0.50) than confidence in incorrect trials (mean = 2.68, s.d. = 0.83). This general trend was also evident in the 2F condition, with confidence slightly higher in correct (mean = 3.16, s.d. = 0.68) compared with incorrect trials (mean = 3.02, s.d. = 0.68). These trends held for unisensory trials in the auditory domain, with confidence being much higher for correct compared with incorrect trials in the 1B (correct: mean = 3.73, s.d. = 0.38; incorrect: mean = 2.56, s.d. = 1.27) and 2B (correct: mean = 3.70, s.d. = 0.41; incorrect: mean = 2.85, s.d. = 1.06) conditions.

With multisensory conditions, some interesting trends emerged. For the trials with congruent multisensory information, as in the unisensory conditions, correct trials exhibited higher confidence than incorrect trials. This was true for not only the 1F1B condition (correct: mean = 3.42, s.d. = 0.52; incorrect: mean = 2.17, s.d. = 0.95), but also the 2F2B condition (correct: mean = 3.27, s.d. = 0.58; incorrect: mean = 2.35, s.d. = 0.83). However, in the 1F2B condition, correct trials actually had slightly lower confidence than incorrect trials (correct: mean = 2.65, s.d. = 0.72; incorrect: mean = 3.06, s.d. = 0.66; *W* = 252.5, *p* = 0.01), and in the 2F1B condition, correct trials again had slightly lower confidence than incorrect trials (correct: mean = 2.84, s.d. = 0.61; incorrect: mean = 3.13, s.d. = 0.67; *W* = 177, *p* < 0.01). In other words, when subjects (incorrectly) integrated the stimuli, their confidence was slightly higher than when they (correctly) segregated the stimuli in these illusion conditions.

## 4. Discussion

In this investigation, we aimed to study if metacognitive confidence judgements differed between congruent and integrated (illusory) multisensory stimuli, and if confidence differed between these two conditions when the reported percept was the same. Using the sound-induced flash illusion, we were able to successfully induce both the fission and fusion illusions, to facilitate comparison with congruent bisensory conditions. Our results showed that, overall, confidence judgements were highest for congruent conditions and lowest for incongruent, illusory conditions. Further exploration showed that under conditions with matched reports, confidence was again higher for congruent conditions, and lower for illusory conditions. Together, these results support the conclusion that metacognition can distinguish between congruent and illusory multisensory information. Finally, additional analyses showed that, in general, correct trials had higher confidence than incorrect trials in many conditions (including unisensory visual, unisensory auditory, and congruent bisensory conditions), but for multisensory conditions with mismatches between the number of beeps and flashes (the 1F2B and 2F1B conditions), confidence was actually lower for correct trials compared with incorrect trials, revealing that the incorrect (integrated) trials had higher average confidence than correct (segregated) trials.

These findings demonstrate the importance of needing to tease apart metacognitive differences across three types of multisensory processes: congruent multisensory perception, integrated multisensory perception, and segregated multisensory perception when distinct multisensory signals are successfully kept separate from one another. Currently, it is unknown if metacognition across these three processes shows similar profiles across different types of multisensory tasks. Our results provide one step towards better understanding this phenomenon. Further, our findings stress the importance of future multisensory research to distinguish between two different metacognitive measures: metacognitive bias, and metacognitive sensitivity. Technically, metacognitive bias is defined as having relatively high or low confidence at a given performance level, while metacognitive sensitivity is defined by how effectively confidence judgements can distinguish between correct and incorrect judgements [4]. Moving forward, measures such as type 2 receiver operating characteristic (ROC) can be employed to effectively evaluate metacognitive sensitivity in multisensory tasks across an array of paradigms and conditions.

Recent work on perceptual reality monitoring has highlighted the important role that metacognition may play in distinguishing between different sources of information, such as being aware of the differences between perceived and imagined sources of information in the environment [19]. According to this work, higher-order cortical regions such as prefrontal cortex may play an important role in making these types of source attribution judgements [48,49], as metacognition and reality monitoring may rely upon shared neural mechanisms [20,50]. Interestingly, in the multisensory literature, inferences about the source(s) of sensory information have also recently been conceived as a hierarchical process [51], with early sensory regions associated with unisensory estimates, and higher-order cortical regions associated with encoding uncertainty about the causal structure (i.e. the sources that give rise to sensory information) of the world [52]. The authors of of [51,52] noted that the prefrontal cortex has previously been implicated in computations related to the causal structure [53,54], which raises an interesting question: are there shared neural mechanisms that support source monitoring in general, whether it be due to distinguishing between perception and imagination, or distinguishing between different sources of multisensory information in the environment?

While conjecture on this point is purely speculative (for now), it appears that the brain's ability to distinguish between different sources of sensory information is an ability that likely extends across domains and tasks. For example, our research group recently demonstrated that confidence is higher for congruent multisensory information compared with integrated multisensory information, even under conditions with matched reports. Kimmet *et al*. [55] used an audiovisual speech (McGurk) task and demonstrated that, even when the reported syllable was the same, average confidence values were higher for congruent McGurk stimuli compared with integrated McGurk stimuli, for an array of audiovisual syllable combinations.

Thus, despite a wealth of multisensory literature referring to integrated multisensory experiences as ‘illusions’ [56–60], an interesting trend is emerging: participants often know when experiences are integrated (or illusory), and when they come from a single source in the environment.

Thus, we can return to the question we raised in the introduction about the metacognitive profile for integrated information: while two decades of research on multisensory integration have emphasized that integration results in an increase in precision in the combined estimate of multisensory information [30–32,61], it appears that metacognitive confidence in integrated estimates of sensory properties is lower than for congruent multisensory information from a single source.

One can also wonder if these metacognitive differences raise any interesting questions about the phenomenology of these illusions. For audiovisual speech illusions like the McGurk illusions, integrated audiovisual speech seems to be a purely ‘perceptual’ effect; while conflict between integrated auditory and visual information may result in confidence being lower than for congruent stimulation, the McGurk illusion seems profoundly perceptual in nature. However, for other types of multisensory illusions, there may be more reason for questioning and investigating the phenomenological nature of reported effects. For example, in the sound-induced flash illusion, research has shown that observers are able to distinguish between illusory flashes and real flashes [46]. Similarly, in other illusions such as the spatial ventriloquist illusion, it would be interesting to see if observers could distinguish between auditory stimulation at one specific location and integrated audiovisual information that results in the auditory localization occurring at that same location (i.e. could they accurately identify that the spatial position is different across the two scenarios?) [18]. Rich debates have permeated the multisensory literature in the last decade regarding whether multisensory judgements are best reflected by truly perceptual effects or (cognitive) response biases [62–64], which is a non-trivial issue that has extended to other perceptual phenomena [65–67]. While additional evidence can be found to support the notion of specific effects like the sound-induced flash illusion being truly perceptual (e.g. by exhibiting feedback resistance, as in [68], or showing correlates in early cortical areas [69,70]), further work may be needed to illuminate how metacognitive differences across conditions relate to phenomenology.

Lurking beneath these issues is a particularly difficult issue to resolve: if multisensory perception is indeed Bayesian in nature [37,71–75], then multisensory perception is influenced by priors. How can we determine which influences on priors are *cognitive* in nature, versus *perceptual* in nature? Sensory experience can be instructive in many ways; for example, the light-from-above prior can be altered by sensory experience and influence perceptual judgements in later trials [76]. But sensory experience can also be informative in regards to stimulus frequencies or sensory rewards, which influence perceptual judgements via more ‘cognitive’ influences [77]. Presently, there may not currently be a clear-cut way to determine which influences change phenomenology, and which simply alter perceptual decision making. While metacognitive or ‘type 2’ judgements may provide some insights into this question [78], more work is needed to further parse these issues.

Overall, we think that the next decade of multisensory research will be especially fruitful, and that the study of metacognition within multisensory paradigms will yield many insights into the nature of the neural basis of metacognition and the function(s) that it serves. One hypothesis regarding the purpose of metacognition relates to information-seeking [79–81], in that specific metacognitive signals may drive further exploratory or information-gathering behaviours. Specifically, metacognition may link to information-seeking via some type of inverted U-function, where extremely high or extremely low confidence is associated with little information-seeking (if you know what something is, or information comes from an extremely noisy source, it may not be worthwhile to pursue further information), but intermediate levels of confidence may be linked to greater information-seeking to resolve ambiguities in stimuli. In this sense, perhaps lower levels of confidence for integrated multisensory stimuli could drive further information-seeking to determine whether the integrated signals truly came from a single source, or whether further exploration could lead to a more accurate inference about multiple sources of information being present. Moving forward, research could pursue the metacognitive profile for multisensory judgements across an array of difficulty levels and in environments where participants can make choices about how long to sample information, to determine if these hypotheses are correct. We think that in order to fully understand the brain's capacity for metacognition, multisensory paradigms must be used, and that better understanding the profile of metacognition in well-known illusions in the field represents a solid foundation to build upon.

## Data Availability

Our data are available from the Open Science Framework website: https://osf.io/p6f3k/. If you have any further questions about the data or files used to run (or analyse) this experiment, please contact Brian Odegaard: bodegaard@ufl.edu.
